# Cannabidiol Protects against the Reinstatement of Oxycodone-Induced Conditioned Place Preference in Adolescent Male but Not Female Rats: The Role of MOR and CB1R

**DOI:** 10.3390/ijms25126651

**Published:** 2024-06-17

**Authors:** Justyna Socha, Pawel Grochecki, Marta Marszalek-Grabska, Aleksandra Skrok, Irena Smaga, Tymoteusz Slowik, Wojciech Prazmo, Robert Kotlinski, Malgorzata Filip, Jolanta H. Kotlinska

**Affiliations:** 1 Department of Pharmacology and Pharmacodynamics, Medical University of Lublin, Chodzki 4a, 20-093 Lublin, Poland; justynasocha97@gmail.com (J.S.); pawelgrochecki@umlub.pl (P.G.); ola234s@wp.pl (A.S.); 2 Department of Experimental and Clinical Pharmacology, Medical University, Jaczewskiego 8b, 20-090 Lublin, Poland; marta.marszalek-grabska@umlub.pl; 3 Department of Drug Addiction Pharmacology, Maj Institute of Pharmacology Polish Academy of Sciences, Smetna 12, 31-343 Krakow, Poland; smaga@if-pan.krakow.pl (I.S.); mal.fil@if-pan.krakow.pl (M.F.); 4 Experimental Medicine Center, Medical University, Jaczewskiego 8, 20-090 Lublin, Poland; tymoteusz.slowik@umlub.pl; 5 Breast Surgery Department, Provincial Specialist Hospital, Al. Krasnicka 100, 20-718 Lublin, Poland; prazmo@prazmo.com.pl; 6 Clinical Department of Cardiac Surgery, University of Rzeszow, Lwowska 60, 35-301 Rzeszow, Poland; robert.kotlinska@icloud.com

**Keywords:** oxycodone, cannabidiol, reward, conditioned place preference, MOR, CB1R, adolescent rats

## Abstract

Cannabidiol (CBD), a phytocannabinoid, appeared to satisfy several criteria for a safe approach to preventing drug-taking behavior, including opioids. However, most successful preclinical and clinical results come from studies in adult males. We examined whether systemic injections of CBD (10 mg/kg, i.p.) during extinction of oxycodone (OXY, 3 mg/kg, i.p.) induced conditioned place preference (CPP) could attenuate the reinstatement of CPP brought about by OXY (1.5 mg/kg, i.p.) priming in adolescent rats of both sexes, and whether this effect is sex dependent. Accordingly, a priming dose of OXY produced reinstatement of the previously extinguished CPP in males and females. In both sexes, this effect was linked to locomotor sensitization that was blunted by CBD pretreatments. However, CBD was able to prevent the reinstatement of OXY-induced CPP only in adolescent males and this outcome was associated with an increased cannabinoid 1 receptor (CB1R) and a decreased mu opioid receptor (MOR) expression in the prefrontal cortex (PFC). The reinstatement of CCP in females was associated with a decreased MOR expression, but no changes were detected in CB1R in the hippocampus (HIP). Moreover, CBD administration during extinction significantly potentialized the reduced MOR expression in the PFC of males and showed a tendency to potentiate the reduced MOR in the HIP of females. Additionally, CBD reversed OXY-induced deficits of recognition memory only in males. These results suggest that CBD could reduce reinstatement to OXY seeking after a period of abstinence in adolescent male but not female rats. However, more investigation is required.

## 1. Introduction

The growing abuse of both prescription and illegal opioids has led to a nationwide healthcare crisis in the United States. Oxycodone (OXY) is one of the most prescribed and abused opioid painkillers and carries a high risk of opioid dependence and misuse [1,2]. Published data show that OXY is very often the first opioid abused in adolescents enrolled in substance use disorders [3]. It is a semi-synthetic opioid analgesic processed from thebaine; a lesser constituent found in opium [4]. It is used to treat both acute and chronic pain and has become the most prescribed opioid painkiller in some countries, surpassing morphine [5]. Pharmacologically, OXY acts as a moderately selective agonist for the mu opioid receptor (MOR). It exhibits a lower affinity for MOR compared to morphine and demonstrates minimal affinity for delta (DOR) and kappa (KOR) opioid receptors [6]. OXY crosses the blood–brain barrier with a permeability that is seven times greater than that of morphine. It also has a quicker onset and a longer duration of action compared to morphine [6], while causing fewer side effects [7]. Due to its potent effects on the brain’s reward system, oxycodone is among the most abused drugs today.

The opioid crisis has brought attention to the difficulties in treating opioid use disorder (OUD), primarily because existing medications mainly consist of MOR agonist substitution pharmacotherapies like methadone and buprenorphine [8,9]. The significant stigma and strict governmental regulations surrounding these pharmacotherapies, attributed to their addictive nature and diversion to the black market, create additional barriers to clinical care and access. As a result, these medications are not fully utilized in addressing the needs of millions of people with OUD [10,11]. The treatment gap for the considerable number of patients with OUD underscores the pressing necessity for innovative therapeutic approaches that diverge from targeting the MOR. Emerging evidence indicates that the reward and reinforcement mechanisms of opioids necessitate interaction between the endocannabinoid (ECS) and opioid systems within the brain [12,13,14]. For example, lower levels of opioid seeking-, reinforcement-, dependence-, and relapse-like behaviors accompany the attenuation of cannabinoid receptors 1 (CB1R) signaling [15,16,17,18,19].

Of note, the ECS has a fundamental role in the signaling of rewarding events [20,21]. ECS (2-arachidonoly glycerol, 2-AG; anandamide, AEA) and cannabinoid receptors (CB1R, CB2R) are widely expressed in brain regions of the mesocorticolimbic system, and it is thought that they can influence dopaminergic signaling within this pathway [22,23,24,25]. The ECS also appears to be involved in the plasticity of the mesocorticolimbic system, which is essential for the development of adaptive changes that lead to drug dependence [26,27]. Currently, modulating the ECS has been identified as a potential target for developing strategies to treat drug addiction [28]. Regrettably, the clinical application of direct (orthosteric) CB1R antagonists (inverse agonists) is restricted due to adverse side effects, such as depression, anxiety, and suicidal thoughts [29,30,31].

Cannabidiol (CBD) appears to satisfy several criteria for a safe approach to preventing drug-taking behavior. CBD is a phytocannabinoid constituent of the *Cannabis sativa* plant devoid of addictive effects [32]. The pharmacology of CBD is not completely understood because this compound has multiple mechanisms of action. It influences several receptors and systems, including ECS [33], serotonergic [34], and opioid [35] systems, among others [see review [36]]. CBD, moreover, is a partial agonist of the D2 receptors, and stimulates TRPV1 and 5-HT1A receptors [37,38,39]. Within the ECS, CBD has a low affinity for CB1R and CB2R [40], even though CBD exerts a negative allosteric modulation effect on both receptors [41]. In addition, CBD has been shown to reduce the hydrolysis of the endocannabinoid AEA through its interaction with fatty acid amide hydrolase (FAAH) [41,42]. Cumulative evidence suggests that CBD lacks abuse potential, as it does not produce rewarding effects and does not trigger withdrawal symptoms following repeated administration [43,44]. In addition, CBD is relatively long-lasting [45], and it appears to reduce anxiety, which is common among people with substance use disorders [46]. The “anti-addictive” actions of CBD have been described in some substance use disorders, including opioid, alcohol, and psychostimulants, mostly in adult male animals [28,47,48].

The drug-induced conditioned place preference (CPP) paradigm commonly used to assess the rewarding properties of addictive substances involves associating the pleasurable effects of these drugs with a specific environment through classical conditioning. Over time, this environment acquires and retains secondary motivational significance, a process that is integral to addiction research [49]. Extinguishing this preference and then later reinstating it can be prompted by drug priming or stress, triggering the retrieval of the rewarding memories associated with the drug [49,50]. The objective of the present study was to investigate the influence of CBD on extinction and reinstatement of OXY-induced CPP in adolescent male and female rats. The reinstatement was induced by priming dose of OXY. Furthermore, locomotor activity was evaluated during the CPP expression and reinstatement phase. Here, we also applied the novel object recognition (NOR) task to determine if deficits in declarative memory have an impact on the CPP reinstatement in rats. Finally, we estimated the role of MOR and CB1R in OXY prime-induced CPP reinstatement in the prefrontal cortex (PFC) and hippocampus (HIP), the brain structures involved in drug addiction in adolescents of both sexes [51,52].

## 2. Results

### 2.1. OXY-Induced Significant CPP

**Figure 1:**
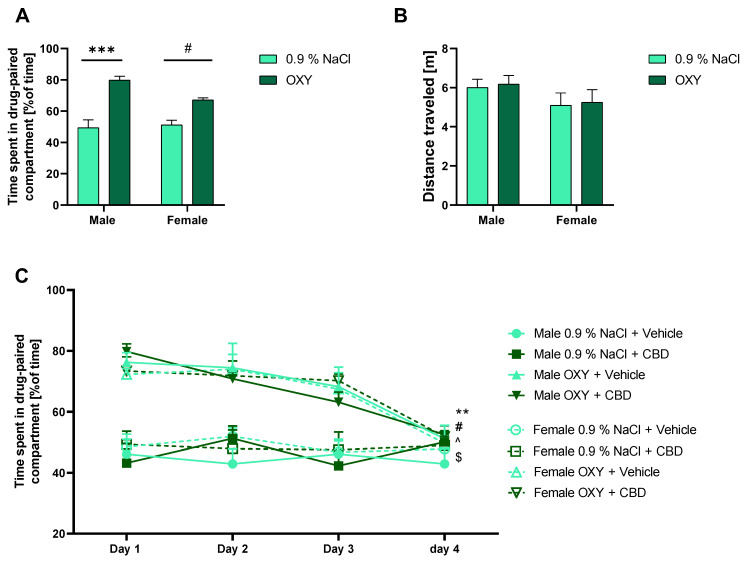
The effect of repeated OXY administration on rat behavior (male and female) in the CPP paradigm. (**A**) OXY increased time spent in the drug-paired compartment during the CPP expression test, *** *p* < 0.001; # *p* < 0.05; (**B**) OXY did not have impact on locomotor activity; (**C**) CBD administration did not accelerate extinction of OXY-induced CPP. Comparison between the 1st day and 4th: ** *p* < 0.01 vs. male OXY + CBD; # *p* < 0.05 vs. male OXY + vehicle; ^ *p* < 0.05 vs. female OXY + CBD; $ *p* < 0.05 vs. female OXY + vehicle. All data are expressed as mean ± SEM.

This study investigated the ability of OXY to induce rewarding effects in adolescent rats of both sexes. The CPP expression test revealed that OXY given during the conditioning phase of the CPP task produced a significant rewarding effect in both male and female rats. The statistical analysis (two-way ANOVA) of time spent in the drug-paired compartment showed a significant effect of OXY [F (1, 20) = 51.67; *p* < 0.001], but no significant effect of sex [F (1, 20) = 2.833; *p* > 0.05], and a significant effect of interaction of these factors [F (1, 20) = 5028; *p* < 0.05]. Tukey’s multiple comparisons test showed statistically significant differences in time spent in OXY and 0.9% NaCl paired compartment both in male (*p* < 0.001) and female rats (*p* < 0.05) (Figure 1A). Moreover, neither OXY treatment ([F (1, 20) = 0.09285; *p* > 0.05]) nor sex of rats ([F (1, 20) = 2.838; *p* > 0.05]) affected the distance traveled by tested rats during the CPP expression (Figure 1B).

### 2.2. Extinction of CPP

After 4 days of CPP extinction, the tested animals did not show any place preference, and CBD administration (once daily) during this period did not accelerate the extinction. In the male rats, the three-way ANOVA showed a significant effect of time [F (3, 80) = 5.610; *p* < 0.01], OXY [F (1, 80) = 84.94; *p* < 0.001] and time × OXY interaction [F (3, 80) = 6.462; *p* < 0.001], but no significant effect of CBD [F (1, 80) = 0.07279; *p* > 0.05]. In the female rats, the three-way ANOVA indicated a significant effect of time [F (3, 80) = 6.476; *p* < 0.001], OXY [F (1, 80) = 67.98; *p* < 0.001] and time × OXY interaction [F (3, 80) = 5716; *p* < 0.01], but no significant effect of CBD [F (1, 80) = 0.02489; *p* > 0.05]. Furthermore, comparisons between groups via Tukey’s test showed significant differences in time spent in drug-paired compartment between days 1 vs. 4 of extinction in male (*p* < 0.01) and female (*p* < 0.05) CBD-treated rats, as well as male (*p* < 0.05) and female (*p* < 0.05) vehicle-treated rats (Figure 1C).

### 2.3. CBD Prevented Reinstatement of OXY-Induced CPP in Male, but Not Female Rats

**Figure 2:**
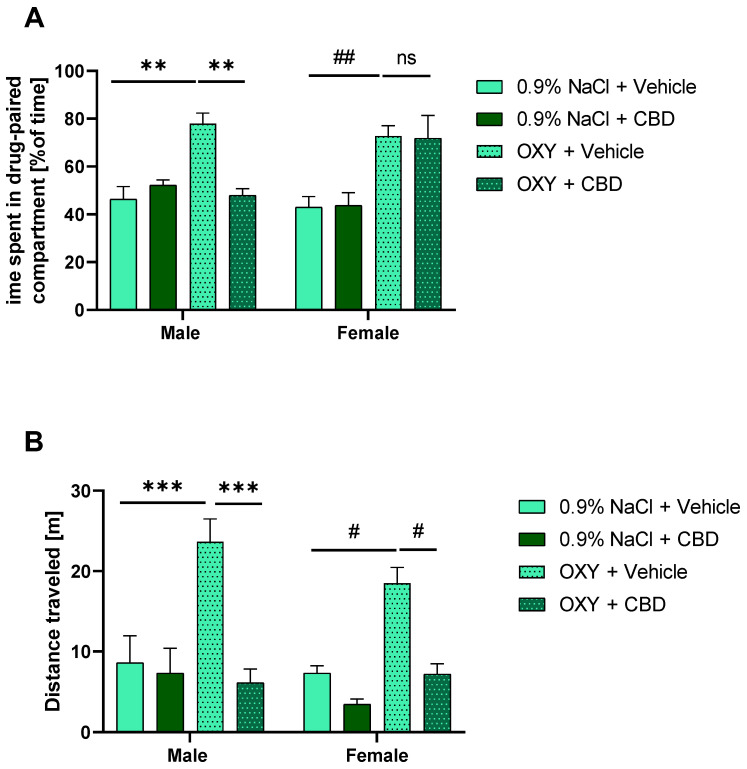
Effect of CBD given during extinction on reinstatement of OXY-induced CPP. (**A**) OXY-induced CPP was reinstated by OXY priming dose both in males and females; however, repeated CBD treatment during extinction was able to diminish this effect only in male rats, ** *p* < 0.01, ## *p* < 0.01, ns—not significant; (**B**) OXY priming induced locomotor sensitization in OXY-treated rats, CBD diminished this effect in both male and female rats (with a stronger effect in males) *** *p* < 0.001; # *p* < 0.05. All data are expressed as mean ± SEM.

Our results revealed that repeated CBD administration during the extinction period prevented the reinstatement of the CPP induced by the OXY priming dose only in male rats, which means that there are sex differences in CBD effect in adolescent rats. The three-way ANOVA showed a significant effect of OXY [F (1, 40) = 33.08; *p* < 0.001], but no significant effect of sex [F (1, 40) = 0.2177; *p* > 0.05] nor CBD [F (1, 40) = 2.682; *p* > 0.05]. Moreover, the three-way ANOVA indicated a significant effect of OXY × sex [F (1, 40) = 4.269; *p* < 0.05], OXY × CBD [F (1, 40) = 6.467; *p* < 0.05], and OXY × sex × CBD [F (1, 40) = 5.351] interactions. Tukey’s multiple comparisons test showed statistically significant differences in time spent in drug-paired compartment between 0.9% NaCl + Vehicle vs. OXY + Vehicle (*p* < 0.01) and OXY + Vehicle vs. OXY + CBD male rats (*p* < 0.01]. In female rats, Tukey’s test showed statistically significant differences between 0.9% NaCl + Vehicle vs. OXY + Vehicle (*p* < 0.05), but CBD did not reverse this effect (Figure 2A). CBD alone did not affect the time spent in the drug-paired chamber.

### 2.4. CBD Prevented Locomotor Hyperactivity Induced by Priming Dose of OXY

Repeated administration of CBD during the extinction phase of CPP inhibited OXY priming dose-induced hyperactivity on the reinstatement day, and effectively prevented the manifestation of behavioral sensitization produced by repeated OXY treatment. The three-way ANOVA showed a significant effect of OXY [F (1, 38) = 20.41; *p* < 0.001], CBD [F (1, 38) = 28.54; *p* < 0.001], and OXY × CBD interaction [F (1, 38) = 13.84; *p*< 0.001]. However, the effect of sex [F (1, 38) = 2.086] and other interactions were not significant. Tukey’s multiple comparisons test showed statistically significant differences between 0.9% NaCl + Vehicle vs. OXY + Vehicle (*p* < 0.001) and OXY + Vehicle vs. OXY + CBD male rats (*p* < 0.001). In female rats, Tukey’s multiple comparisons test showed statistically significant differences between 0.9% NaCl + Vehicle vs. OXY + Vehicle (*p* < 0.05) and OXY + Vehicle vs. OXY + CBD (*p* < 0.05) groups (Figure 2B). Thus, our findings show that CBD had a preventive effect on hyperactivity induced by a priming dose of OXY, and this effect was more pronounced in males than females.

### 2.5. CBD Reversed Recognition Memory Impairment Induced by OXY in Male, but Not Female Rats

**Figure 3:**
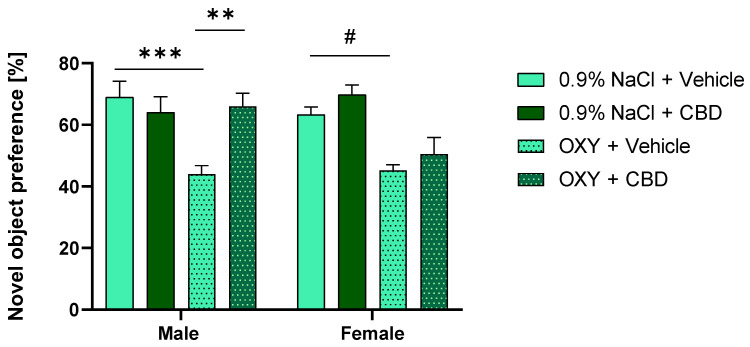
Effect of repeated OXY and CBD administration on short-term memory in NOR task in male and female adolescent rats. OXY administration induced declarative memory deficits in NOR task both in males and females, and CBD treatment was able to prevent this effect only in male rats. *** *p* < 0.001; ** *p* < 0.01; # *p* < 0.05. All data are expressed as mean ± SEM.

Adolescent animals were subjected to the NOR task to evaluate whether memory deficits have an impact on the OXY prime-induced outcome from the OXY-induced reinstatement of CPP. Our findings demonstrated that repeated OXY administration diminished declarative memory in the NOR task in both male and female adolescent rats; however, repeated CBD treatment was able to reverse this effect in male, but not in female rats. The three-way ANOVA showed a significant effect of OXY [F (1, 40) = 28.62; *p* < 0.001] and CBD [F (1, 40) = 6.457; *p* < 0.05], but no significant effect of sex [F (1, 40) = 1.576; *p* > 0.05]. Moreover, the three-way ANOVA indicated a significant effect of OXY × CBD [F (1, 40) = 1.605; *p* < 0.05] and OXY × sex × CBD [F (1, 40) = 6184; *p* < 0.05] interactions. Tukey’s test showed significant differences in preference score between 0.9% NaCl + Vehicle vs. OXY + Vehicle (*p* < 0.001) in male rats, and the same effect was observed in females (*p* < 0.05); however, this effect was reversed by CBD only in male (*p* < 0.01) and not in female rats (*p* > 0.05) (Figure 3).

### 2.6. Adolescent OXY and CBD Exposure Differentially Modify MOR and CB1R Expression in the PFC and HIP in Adolescent Male and Female Rats

To assess the correlation between behavioral and neurochemical changes induced by OXY, as well as the impact of CBD on these changes, we examined (using ELISA) the expression of MOR and CB1R in the PFC and HIP, structures involved in memory processes.

**Figure 4:**
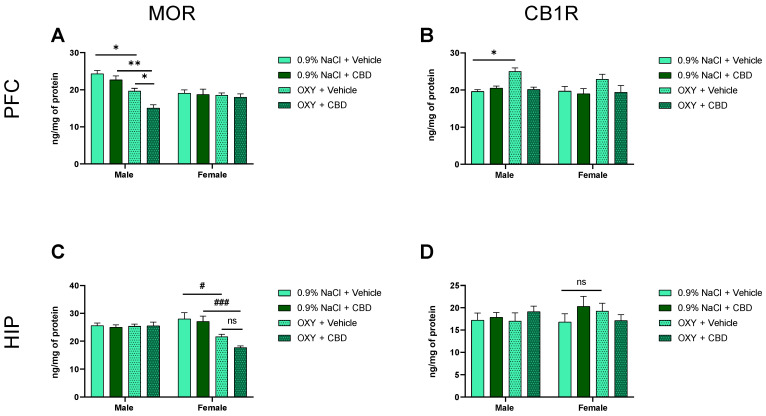
Effect of OXY and CBD administration on the expression of MOR and CB1R in the PFC (**A**,**B**) and HIP (**C**,**D**) in male and female adolescent rats. OXY decreased MOR expression in the PFC of males (**A**) and in the HIP of females (**C**), but increased CB1 expression in the PFC of males (**B**). Moreover, CBD potentialized the effect of OXY on MOR in the PFC of males (**A**), ### *p* < 0.001; ** *p* < 0.01; * *p* < 0.05; # *p* < 0.05, but not females (**C**), ns—not significant. All data are expressed as mean ± SEM.

Data from the three-way ANOVA indicated a significant effect of OXY [F (1, 40) = 25.21; *p* < 0.001], sex [F (1, 40) = 7.524; *p* < 0.01], CBD [F (1, 40) = 7.274; *p* < 0.05], and OXY × sex interaction [F (1, 40) = 16.81; *p* < 0.001] on MOR expression in the PFC of rats. Tukey’s multiple comparisons test showed statistically significant differences between 0.9% NaCl + Vehicle vs. OXY + Vehicle (*p* < 0.05), OXY + Vehicle vs. OXY + CBD (*p* < 0.05), and between 0.9%/CBD vs. OXY + CBD (*p* > 0.05) in male rats (Figure 4A). These results suggest that OXY-induced changes in MOR in the PFC were seen only in male rats and CBD potentiated this effect. However, in females, OXY-induced alterations in the MOR expression only in the HIP, and CBD treatment had no impact on these changes. The three-way ANOVA indicated a significant effect of OXY [F (1, 40) = 17.29; *p* < 0.001] and OXY × sex interaction [F (1, 40) = 18.57; *p* < 0.001] on MOR expression in the HIP. However, the effects of sex [F (1, 40) = 3.618; *p* > 0.05] and CBD [F (1, 40) = 2.176; *p* > 0.05] were not significant. Tukey’s test showed statistically significant differences between 0.9% NaCl + Vehicle vs. OXY + Vehicle (*p* < 0.05) and 0.9% NaCl/CBD vs. OXY + CBD (*p* < 0.01) in female rats (Figure 4C).

Furthermore, repeated OXY administration enhanced the expression of CB1R in the PFC of male, but not female rats. Subsequent administration of CBD did not change this effect. The three-way ANOVA indicated a significant effect of OXY [F (1, 40) = 7.527: *p* < 0.01], CBD [F (1, 40) = 6.646; *p* < 0.05], and OXY × CBD interaction on CB1R expression in the PFC of rats. Tukey’s multiple comparisons test showed that OXY significantly increased CB1R expression in male rats (*p* < 0.01), while in females, this effect was not significant (*p* > 0.05) (Figure 4B). In addition, no significant changes were seen in the hippocampal expression of CB1R in male and female adolescent rats. ANOVA analysis did not reveal a significant effect of OXY [F (1, 40) = 0.008814; *p* > 0.05], sex [F (1, 40) = 0.2507; *p* > 0.05], nor CBD [F (1, 40) = 0.7406; *p* > 0.05] on CB1R expression in the HIP of the tested animals (Figure 4D).

## 3. Discussion

The findings presented in this study offer insights into the impact of CBD administration on drug seeking behavior associated with OXY addiction in adolescent male and female rats. Specifically, CBD was effective in preventing OXY prime-induced CPP reinstatement, locomotor sensitization, and memory deficits associated with OXY injections in male adolescent rats. In addition, CBD had an impact on MOR and CB1R expression in the PFC of male rats. In female rats, CBD failed to affect behavioral outcomes associated with OXY injections, except for locomotor sensitization. These data suggest that there are sex differences in CBD effects on OXY-induced behavior associated with addiction in adolescent male and female rats.

### 3.1. Effects of CBD on Drug Prime-Induced CPP Reinstatement

In our study, adolescent male and female rats both developed CPP induced by OXY (3 mg/kg) in the contextual assay, despite males initially showing a higher preference than females for the drug compartment. These results are consistent with previous reports wherein OXY, at the same dose, induced CPP in adult male [50,53] and female rats [54]. In the present study, CBD, given prior to each extinction trial, did not accelerate the extinction of conditioned behavior in male and female rats. These data are in accordance with a very recent investigation conducted in adult male mice, in which CBD given prior to each extinction trial did not accelerate CPP extinction, but it blocked the priming-induced reinstatement of cocaine CPP [55]. Other studies indicate that CBD given intracerebroventricularly (icv) prior to every extinction session accelerated the extinction and prevented the reinstatement of extinguished methylphenidate-induced CPP by methylphenidate priming dose in adult male rats [56]. Still other research shows that in male adult Wistar rats, the injection of CBD (10 mg/kg, i.p.) prevented the development of CPP behavior, whether it was reinstated by a priming dose of morphine or through exposure to stress [57]. In male adult C57BL/6J mice, administering the same dose of CBD also significantly lowered the development of morphine-induced conditioned place preference (CPP) [58]. In our study, CBD given prior to each extinction trial prevented the CPP reinstatement induced by OXY priming dose in adolescent male, but not female rats. Thus, our results support previously published data in male animals that CBD can reduce cue-induced reinstatement of opioid (drug) seeking behavior after extinction. The rewarding effects of OXY are primarily mediated by its action on the MOR in the mesolimbic and nigrostriatal pathways [59]. Thus, one of the mechanisms of action of CBD is that it can lead to a reduction in dopaminergic transmission [37,60,61], which might cause anti-reward effects. However, it is difficult to explain why such effect is observed only in male animals.

### 3.2. Impact of CBD on Locomotor Activity in Drug Prime-Induced CPP Reinstatement

The locomotor responses triggered by addictive substances offer a method to examine both drug sensitivity issues and the neuroplastic changes caused by drug use. In the current study, locomotor response of animals did not differ during expression of CPP when animals were free of drugs; however, locomotor activity of animals was significantly increased during the reinstatement session after a priming dose of OXY (1.5 mg/kg). The increase of locomotor activity after intermittent administration was observed in both sexes in the OXY-treated group (vs. control) that received a priming dose of OXY. Such an increase of locomotor activity in previously OXY-treated rats implies an increase in sensitivity to OXY. Furthermore, repeated treatment with CBD did not change the locomotor effect of an acute dose of OXY (1.5 mg/kg) in both sexes. Thus, the current findings indicate that adolescent rats can develop locomotor sensitization to OXY, potentially context-dependent, reflecting neuroplasticity in response to repeated exposure to MOR agonists. These results underscore the susceptibility of adolescent individuals to OXY dependence. We find that male adolescent rats exhibit higher preference for OXY-paired context in the reinstatement procedure than female rats. Moreover, male animals display greater locomotor sensitization than females. However, other authors showed [62] opposing relationships between the locomotor effects of morphine and CPP in female vs. male adult mice.

In our study, this effect of OXY was ameliorated by prior CBD treatment during CPP extinction in both sexes. Previous research has outlined the role of CB1R in influencing the pharmacological effects of various addictive substances. Specifically, CB1R activation has been identified as pivotal in the development of opioid-induced locomotor sensitization. Studies have shown that mice lacking CB1R (CB1R KO) did not exhibit sensitization to morphine [63]. Furthermore, other investigations have indicated that the CB1R receptor antagonist, rimonabant, effectively prevented the manifestation of behavioral sensitization induced by morphine, with its effectiveness being dependent on the context in adult female mice when given in a drug-associated environment, but not in their home cage [64]. In our study, CBD, as a negative allosteric CB1R modulator [65] reduced OXY-induced locomotor sensitization in both sexes but failed to prevent the CPP reinstatement in female rats. The findings imply that the impacts of repeated CBD exposure on reward and motor functions can be separated in adolescent female rats. Indeed, they suggest that diminishing cannabinoid activity might forecast changes in motor function that are distinct from alterations in reward perception.

### 3.3. Impact of CBD on Learning and Memory Deficits in OXY-Treated Adolescent Rats

After exposure to drug-associated cues, drug reward memory can enter two opposing processes: reconsolidation and extinction [66]. Both are promising approaches to regulating drug reward memory and preventing relapse. Published data show that CBD given just after reactivation CPP sessions impairs reconsolidation (a process during which original memory could be updated) of morphine-reward memory lasting at least 2 weeks, as well as reinstatement of morphine-induced CPP and conditioned place aversion (CPA) induced by naltrexone administration in adult male Wistar rats [57]. In our study, CBD treatment before every extinction session (the formation of new inhibitory learning rather than an erasure of the original (drug) memory) [67] did not accelerate the extinction of OXY-induced CPP in male and female adolescent Wistar rats. Still, despite the lack of a non-extinction control, we observed that CBD reduces the reinstatement of OXY-induced place preference, and this suggests that such reduction was, at least in part, due to the concomitant extinction trial. One of the hypotheses that could explain such outcome is that CBD causes a memory impairment. Therefore, we tested whether CBD affected nonemotional memory by using the NOR task. The experiment was performed on the reinstatement day after an OXY priming dose. Our results revealed that repeated CBD administration did not affect the short-term memory of either novel or familiar objects, but it reversed memory impairment induced by repeated OXY administration in male rats. Considering that pharmacological enhancement of extinction typically relies on substantial reduction of extinction-mediated memory [68,69], and there was no evidence for any such reduction in our present study and other CBD studies [70], it remains unclear if CBD enhanced drug memory extinction in our study.

### 3.4. MOR and CB1R in Drug Prime-Induced CPP Reinstatement

Published data showed that signaling through ECS within the limbic brain regions, including the PFC and HIP, has been demonstrated to profoundly influence the emotional and memory-related processing of motivational signals associated with opioid dependence [71]. Our study supports these data and indicated that the reinstatement of OXY-induced CPP by drug priming was associated with a decrease of MOR and an increase of CB1R expression in the PFC in adolescent male rats, whereas female adolescent rats demonstrated a decrease of MOR and no changes of CB1R expression in the HIP. CBD administration during extinction significantly potentiated the reduction of MOR expression in the PFC of males and only showed tendency to potentiate the reduction of MOR expression in the HIP of females. Thus, although opioid reward and reinforcement require crosstalk between the ECS and opioid system in the brain [12,13,72], our data suggest that in adolescence, there are differences in the response to cannabinoid compounds, especially in females that received opioid.

Adolescence is a critical stage of development characterized by substantial behavioral, morphological, hormonal and neurochemical changes, including alterations in brain regions implicated in the reinforcement and reward mechanisms associated with drugs such as opioids [73]. Our study showed that in adolescent males, the MOR in the PFC is engaged in relapse to OXY CPP reinstatement. Of note, the PFC is involved in decision-making, reward-seeking, and impulsivity [74,75]. Thus, we hypothesize that OXY-induced changes in MOR could be responsible for compulsive intake of abuse drugs and return to addiction. In turn, in females, the MOR in the HIP plays an important role in relapse to OXY prime-induced drug-context reinstatement. The HIP has been implicated in various aspects of drug-related behavior, including the association between context and drug reward-related responses, and the craving and seeking of drugs, particularly in the context of reinstatement and relapse to substance abuse [76]. Moreover, the HIP is crucial for transforming short-term memories into long-term ones, a process thought to contribute to the aberrant learning associated with addiction [77]. Thus, we hypothesize that OXY-induced aberrant learning processes in the HIP are involved in relapse to drug addiction in female adolescent rats in our study.

### 3.5. Limitation and Future Research Direction

Existing research underscores differences between animal sex, age, and strain in susceptibility to addiction. Unlike our study on adolescent females, adult female rats showed a stronger drive to acquire OXY compared to their male counterparts [78,79,80]. Adult female rats exhibited heightened responses during extinction and increased conditioned reinstatement for heroin [81] and fentanyl [82]. Cannabinoids induced greater antinociception, catalepsy, sexual behavior, and anxiety in female rats than in male rats [83], although locomotor and thermoregulatory responses remained similar between sexes [84]. Moreover, intact female rats self-administered cannabinoids at higher rates compared to male rats, whereas ovariectomized females showed less sensitivity to the reinforcing effects of cannabinoids [85]. Consequently, findings suggested that the gonadal hormone estradiol interacts with the ascending telencephalic dopamine system, leading to sex differences in motivated behaviors, including drug-seeking [86]. Given this information, a limitation of our study is the lack of consideration for the hormonal cycle in female rats. There is limited research on sex differences in the impact of CBD on addictive behavior. Nonetheless, similar to our study where CBD did not prevent the reinstatement of OXY-induced CPP in adolescent female rats, other studies have found that CBD did not affect morphine withdrawal syndrome in adult females [87]. Another study limitation is the lack of verification of the role of CB2R in OXY. In fact, recent findings indicate that CB2R agonists disrupted CPP induced by cocaine [88,89] or ethanol [90,91,92]. Additionally, CB2R antagonists have been shown to block the acquisition of nicotine-induced CPP [93,94] and the expression of alcohol-induced CPP [92]. However, this impairment might be due to the aversive properties of CB2 antagonists, as they can induce conditioned place aversion (CPA) [94]. In addicted individuals, CB2R may influence the mesolimbic dopamine system by: (i) affecting microglia and astrocytes, which express CB2R [95,96], or (ii) inhibiting dopamine release in mesolimbic structures [97]. In the hippocampus, CB2R activation is involved in the expression of CPP due to its role in spatial memory recovery [98], leading to the inhibition of cocaine-induced CPP [89]. Furthermore, CB2R can play a role in opioid tolerance and reward-seeking behavior [99]. Research suggests that CB2R may contribute to the effects of CBD, with CBD potentially acting as CB2R antagonist/inverse agonist or partial agonist [60,100]. Thus, future studies should investigate the role of CB2R in the motivational effects induced by OXY.

Apart from unknown the role of CB2R in OXY actions, CBD inhibitory effects on OXY-induced reward might depend on multiple receptor mechanisms depending on the dose [60]. Another possibility is that different receptors could form functional heterodimers or interact at the level of intracellular signaling, meaning that blocking one receptor pharmacologically could affect CBD’s action on other receptors. However, in our experiment, we used only a separate dose of CBD (10 mg/kg), therefore, more research is required to address these issues.

In conclusion, our findings indicate that CBD is effective in preventing OXY seeking behavior after a period of abstinence in adolescent male, but not in female rats. This effect in males appears to be due to the interaction between CB1R and MOR. Further research is needed to determine whether the lack of effect in female adolescent rats is related to gonadal hormone status, dose of CBD, or other mechanisms, such as that, for example, of CB2R. It is important to investigate the mechanisms behind these sex differences, especially since previous research has shown that CBD reduces cue-induced cravings in humans with heroin use disorder [101].

## 4. Materials and Methods

### 4.1. Animals

This study involved 96 Wistar rats of both sexes, which were bred and housed in the vivarium of the Medical University of Lublin, Poland. The vivarium followed a 12 h light/12 h dark cycle, with lights on at 8:00 a.m., and maintained a constant temperature of 22 ± 1 °C and humidity of 55 ± 10%. Throughout the study, the rats had ad libitum access to food and water. The experiments began when the animals were 28 days old (PND28). These were carried out according to the National Institute of Health Guide for the Care and Use of Laboratory Animals, as well as to the European Community Council Directive for Care and Use of Laboratory Animals (86/609/EEC), and was approved by the Local Ethics Committee (30/2023).

### 4.2. Treatment Conditions and Drugs

At the beginning of this study, the animals (96) were divided into 2 sets (for reward and memory test purposes), each subjected into two main groups: 0.9% NaCl and OXY. Next, the 0.9% NaCl group was subdivided into 2 groups: 0.9% NaCl + vehicle (group 1) and 0.9% NaCl + CBD (group 3), while the OXY group was also subdivided into 2 groups: OXY + vehicle (group 2) and OXY + CBD (group 4), each consisting of 12 individuals (6 males and 6 females).

OXY (Norpharma, Copenhagen, Denmark) was diluted in 0.9% NaCl and administered once a day at a dose of 3 mg/kg [53]. CBD (THC Pharm GmbH, Frankfurt, Germany) was administered to animals at a dose of 10 mg/kg (the dose was chosen on the basis of previous work [57]), and was obtained by suspending the substance in a vehicle (1% solution of Tween 80 (Sigma, St. Louis, MO, USA) in 0.9% NaCl). The solutions were prepared just before administration, ex tempore. All substances used in the experiments were administered intraperitoneally (i.p).

### 4.3. Procedures

#### 4.3.1. Conditioned Place Preference (CPP) Test

One set of animals was used in this experiment. CPP is a basic test used to assess the rewarding effect of various addictive substances. It is carried out in the following stages: pre-test, conditioning, place preference test, extinction of preferences, and reinstatement of preferences. The apparatus used to perform this test consists of cages measuring 65 cm × 35 cm × 30 cm, divided into two parts (compartments) and with different colored walls. One compartment had smooth black walls, while the other featured black and white vertical stripes, and both had metal grating floors. These compartments were divided by removable guillotine doors. To ensure cleanliness and to remove any odors, the apparatus underwent thorough cleaning before each test, followed by wiping with dry paper towels. The boxes were placed in a soundproof room with neutral noise masking and subdued 40 lux lighting. Locomotor activity (total distance traveled) and time spent in each chamber were tracked using cameras and computer software (ANY-maze video tracking system 6.3, Stoelting Co. Wood Dale, IL, USA).

##### Habituation Phase and Pre-Test

On PND 28 and 29, the animals were habituated to the apparatus. For this purpose, the rats were placed individually in one of the compartments, and allowed to move freely and explore the full apparatus for 15 min.

A pre-test was performed on PND 30. This part of the study took place before the injections began. Each animal was placed in the apparatus for 15 min with open access to both rooms. In this way, it was tested whether animals showed preferences for staying in specific places. The test showed no such behavior.

##### Conditioning Phase

The conditioning stage lasted 8 days (PND31-38) and aimed to develop place preference after OXY administration [53]. On odd days, animals from groups 1 and 3 were injected with 0.9% NaCl solution, and rats in groups 2 and 4 received OXY at a dose of 3 mg/kg, i.p. (once a day before each conditioning session). The rats were always injected immediately before being placed into the conditioning chamber for 60 min to associate it with a rewarding substance. Some animals were conditioned to the compartment with black walls, and others to the room with white and black stripes. During this stage, the doors in the cage remained closed and the animals were not able to move between compartments. On PND32, 34, 36, and 38, that is, on even-numbered days, each animal received an injection of 0.9% NaCl solution, and then they were placed in the appropriate equipment chamber (opposite to the previous day).

##### CPP Expression Test

Approximately 24 h after the last conditioning session (PND39), the CPP expression test was performed. The animals could move freely between the compartments of the apparatus for 15 min. Time spent in OXY-coupled compartment and distance traveled were measured by video tracking. On this day, the animals did not receive any injection.

##### Extinction Phase

The CPP extinction was performed for the subsequent 4 days (PND40-43) after the CPP test (CPP expression test), and the animals no longer received OXY injections. Half of the rats (groups 3 and 4) were treated with CBD (10 mg/kg, i.p.) and the remaining groups received a vehicle (groups 1 and 2). After injections (once a day, over the 4 days), animals were placed in a random compartment of the apparatus with free access to both chambers. The time spent in compartments was assessed for 60 min to determine whether the place preference had been extinguished. The CPP was considered as extinguished when the time spent in the saline- and drug-paired compartments were similar to those of the pre-test phase.

##### Reinstatement Phase

After CPP extinction, on the last day of the experiment (PND44, corresponding to a human age span of 15–17 [102,103]), each animal received a priming injection of OXY at a dose of 1.5 mg/kg, i.p., and then they were placed in the apparatus with free access to both compartments. The time spent by the rats in a particular compartment and the distance traveled were measured to determine whether place preference was reinstated in the tested animals after a priming dose of OXY.

#### 4.3.2. Novel Object Recognition (NOR) Task

Another set of animals was used in this experiment. Utilization of the NOR task with drug injections identical to those used in the CPP task allowed us to examine whether OXY exposure induced deficits in recognition memory and whether CBD was able to prevent the memory deficits induced by OXY. All animals, regardless of sex and age (PND44), underwent the NOR task within a Plexiglass box measuring 40 cm × 40 cm × 40 cm, illuminated with approximately 20 lux light, in a quiet environment. The animals were acclimated to the apparatus for 30 min before each session of the NOR task. The NOR task consisted of three sessions: (1) habituation, followed by (2) a training session lasting 5 min, and (3) a testing session also lasting 5 min, with a 30 min interval between the training and testing sessions [104,105,106]. During the training session, two identical objects were positioned in diagonal corners of the box. In the subsequent testing session, one of the objects was replaced by a novel object, differing in color and shape from the familiar object. Each animal was separately placed in the center of the box facing one of the remaining empty corners. Both the training and the testing sessions were recorded to provide further analysis of animal behaviors. The evaluation of object recognition was conducted manually by an experimenter who was blind to the experimental conditions, and the results were expressed as a percentage. The objects used were selected based on preliminary studies that demonstrated no inherent preference for any object. Following each session of the NOR task, the animals were returned to their home cages. Immediately before the testing session, each animal received the same priming dose of OXY (1.5 mg/kg, i.p.) as the animals in CPP reinstatement.

#### 4.3.3. Enzyme-Linked Immunosorbent Assay (ELISA)

Right after the CPP procedure, the animals were decapitated, and the dissected brain structures were subjected to biochemical experiments to evaluate the influence of OXY and CBD administration on MOR and CB1R expression. The validation of protein levels involved the use of a Rat MOR ELISA Kit (E1559Ra; Bioassay Technology Laboratory, Shanghai, China) and a Rat CB1R ELISA Kit (E1475Ra; Bioassay Technology Laboratory, China), following the respective manufacturer’s protocols. In brief, rat brain structures were homogenized in cold PBS (pH 7.4) containing protease and phosphatase inhibitors (Sigma-Aldrich, St. Louis, MO, USA), followed by centrifugation at 5000× *g* for 5 min. Protein concentration in the supernates was determined using a bicinchoninic acid assay (BCA) protein assay kit from Serva (Heidelberg, Germany). The absorbance of duplicate samples and standards was measured at a wavelength of λ = 450 nm using a Multiskan Spectrum spectrophotometer (Thermo LabSystems, Philadelphia, PA, USA). Protein concentrations were calculated from standard curves and expressed as ng/mg of protein.

### 4.4. Statistical Analysis

The data obtained were analyzed using Prism v. 8.0.0 for Windows (GraphPad Software, San Diego, CA, USA). Statistical significance of the effects observed in both behavioral and molecular tests was assessed using two- or three-way analysis of variance (ANOVA) with repeated measures, followed by Tukey’s post hoc test. Results were reported as means ± standard errors of means (SEM), with a *p*-value less than 0.05 considered statistically significant for all tests.

## Data Availability

Data are contained within the article.
